# Renoprotective effect of oral rehydration solution III in exertional heatstroke rats

**DOI:** 10.1080/0886022X.2019.1590211

**Published:** 2019-04-03

**Authors:** Yufang Lin, Yanning Zhang

**Affiliations:** Department of Nephrology, General Hospital of Northern Theater Command, Liaoning, China

**Keywords:** Exertional heatstroke, acute kidney injury, neutrophil gelatinase-associated lipocalin, oral rehydration solution, prevention

## Abstract

**Aim**: Exertional heastroke (EHS) can lead to acute kidney injury. Oral rehydration solution III (ORS III), recommended by WHO in 2004, is used to rehydrate children with gastroenteritis. This study aimed to characterize the renoprotective effect of ORS III in EHS rats.

**Methods**: Rats were randomly divided into Group Control, Group EHS, Group EHS + Water, and Group EHS + ORS. Thirty minutes before the experiment, ORS III was orally administrated to Group EHS + ORS, Water was given to Group EHS + Water. Rats from Group EHS, Group EHS + Water and Group EHS + ORS were then forced to run until they fatigued. Core temperature (Tc) was monitored and 40.5 °C was considered as the onset of heatstroke. Serum creatinine (SCr), blood urea nitrogen (BUN) were measured using an automated biochemical analyzer. Serum neutrophil gelatinase-associated lipocalin (NGAL) was measured using an NGAL ELISA Kit. Light microscopy was used for kidney structural analysis.

**Results**: SCr level in Group EHS was no different from Group Control (*p* > .05), while BUN and NGAL levels in Group EHS were higher than Group Control (*p* <.001, *p* < .001). SCr, BUN and NGAL concentrations in group EHS + Water were no different from Group EHS (*p* > .05). SCr, BUN levels in Group EHS + ORS were no different from Group EHS (*p* > .05). But NGAL levels were significant in these two groups (*p* = .012). Renal histopathologies of rats in Group EHS and Group EHS + Water showed flattened lumens filled with eosinophilic materials. The damage was milder in Group EHS + ORS, in which injured tubules showed degeneration of the tubular epithelium and sloughing of the brush border membrane.

**Conclusion**: ORS III could alleviate the kidney injury in EHS rats.

## 1. Introduction

Heatstroke is a life-threatening disease defined as core body temperature value rapidly increases higher than 40 °C, combined with central nervous system abnormalities such as delirium, convulsions, or coma [1]. Exposing in a hot environment will lead to heatstroke, which can be classified into two forms – classical heatstroke (CHS) and exertional heatstroke (EHS). CHS mainly takes place in very young or old people, but EHS was more common in athletes and soldiers when strenuous exercising outdoors [2]. The mortality of EHS was 5–50% in human beings [3]. In the USA, EHS is considered the third leading cause of sudden death in high school athletes [4].In the last century, the global average temperature rose 0.8–0.9 °C, which resulted in higher incidence rate of heatstroke [5]. The clinical spectrum of EHS ranges from a subclinical illness like fatigue, vomit to emergency issues like neurological disorder and kidney injury. Recently, the treatment of heatstroke mainly includes cooling, intravenous hydration, hemodialysis filtration and etc [1,6], but all these therapeutic effects are proved to be unsatisfactory, and little effort has been put into research on the prevention of heatstroke.

EHS can lead to acute kidney injury. The mechanisms between heatstroke and acute kidney injury includes dehydration [7], electrolytes disorder [6], heat cytotoxicity [7], disseminated intravascular coagulation [1], the release of pro-inflammatory cytokines [1,8], and post-renal obstruction by myoglobin [9]. But the specific molecular mechanism has not yet been fully confirmed.

Oral rehydration solution III (ORS III), a low osmolarity ORS, recommended by WHO in 2004 [10], is mainly used to treat children with gastroenteritis [11]. ORS III contains sodium chloride 2.6 g, potassium chloride 1.5 g, sodium citrate 2.9 g, anhydrous glucose 13.5 g. It has an osmotic pressure of 245 mOsm/l [12]. ORS III promotes water absorption via the sodium-dependent glucose co-transporter 1 (SGLT1) in villous cells of the small intestine [12]. The molar ratio of sodium to glucose in ORS III is 1:2, which is ideal for the intestinal absorption of electrolytes and water [13].

Unlike routine kidney biomarkers, serum creatinine (SCr) and blood urea nitrogen (BUN), which are unable to detect the kidney damage at the early stage. Neutrophil gelatinase-associated lipocalin (NGAL), a novel kidney injury biomarker, has proved to be effective in finding early renal kidney lesion [14]. For ICU patients who developed Acute kidney injury (AKI), NGAL had a sensitivity of 91% and a specificity of 95% [15]. In renal ischemia or toxic damage, the NGAL expression notably increases in injured tubular epithelial cells [14,16]. It is one of the earliest, most significantly induced proteins once AKI occurs. It starts to rise 2 h after kidney injury [17], and reaches at highest 6 h later, then falls steadily [18]. It is popularly used in detecting early kidney injury, having a great significance for early diagnosis of AKI.

Hypodration and electrolyte imbalance in EHS will impair overall exercise performance, aggravating the kidney injury caused by heat [19]. Maintaining a full rehydration condition is crucial to EHS patients. In our study, we hypothesized that pretreatment of ORS III would have a renoprotective effect on EHS rats. We examined both the traditional and novel kidney biomarkers to evaluate their diagnostic utility. Moreover, the renal tissues were examined under a light microscope to further assess the renal pathology.

## 2. Method

### 2.1 Animals and ORS III

#### 2.1.1 Animals

All animal protocols were approved by the Animal Ethic Committe of General Hospital of Northern Theater Command. Fifty two male SD rats (200–250 g) were obtained from Changsheng Biotechnology Co. (Shenyang, Liaoning, China). The animals were used under license (License number SCXK 2015–0001). Rats were housed in standard and housed at the animal experiment center in General Hospital of Northern Theater Command on a 12/12 h light-dark cycle. The temperature and humidity of the room were maintained at 25 ± 2 °C and 50 ± 5% relative humidity. They were allowed free access to rat feeds and water.

#### 2.1.2 Groups

Fifty two rats were randomly divided into 4 groups: Group control (*n* = 13); Group EHS (*n* = 13); Group Water + EHS (*n* = 13); Group ORS III + EH (*n* = 13).

#### 2.1.3 Chemicals

ORS III was obtained from Xi’an Anjiang pharmaceutical co. Ltd (Xi’an, Shanxi, China), each bag contained sodium chloride 0.65 g, potassium chloride 0.375 g, sodium citrate 0.725 g, Anhydrous Glucose 3.375 g. It was diluted by pouring the contents into a 250 mL graduated cylinder and added distilled water to a final volume of 250 mL.

### 2.2 Animal training

One week before the experiment, we trained the rats aimed to familiarize them with the environment of the treadmill (Zhongshi technology co. Ltd, Beijing, China) and to make rats adapt to the forced running system. Electrodes were previously designed at the end of each runway. Electrical stimulation was set at 1 mAh to force rats to run. The temperature was 25 ± 2 °C and humidity of the room was 50 ± 5%. On the first day, rats were free to run and explore their surroundings for 15 min. then rats were started to run at an initial speed of 10 m/min for 35 min, On the second day, rats were forced to run at a speed of 11 m/min for 40 min. Speed was increased by 1 m/min and prolonged the exercising time by 5 min per day. On the sixth day, the speed was 15 m/min and the training time was 60 min. Rats were allowed to have a rest on day seven. During this period, all rats were free to water and feeds.

### 2.3 EHS experiment

Following the training session, experiment was performed on the eighth day. One hour prior to the initiation of the EHS protocols, two electromagnetic spectrum heaters (Changle Silicate co. Ltd, Chongqin, China) were put around to pre-warm the treadmill until the local temperature was increased to 37.5 °C. At this temperature, the exertional heat production had the greatest contribution to the overall heat load [3]. Room humidity was 50 ± 5%. 30 min before the experiment, Group Control stayed at the animal lab where the environmental temperature was 25 ± 2 °C and humidity was 50 ± 5%, Group EHS + Water was orally administrated with water (20 mL/kg), and Group EHS + ORS was given an equivalent dosage of ORS III. After measuring the rats’ core temperature (Tc) using an electric thermometer, rats in Group EHS, Group EHS + Water and Group EHS + ORS were then forced to run at a speed of 15 m/min with a 1.0 mAh electrical stimulation until they were fatigue, which was defined as refusal to run for 5 s^3^. At this time, Tc was monitored again and 40.5 °C was considered as the onset of heatstroke [20]. At the end of the protocol, rats were removed from the treadmill to recovery at room temperature.

### 2.4 Measurement of SCr, BUN, NGAL, and blood sampling

The rats were intra-peritoneal anesthetized with 10% chloral hydrate (0.3 mL/100 g) six hours post-presentation. Blood was obtained from the inferior vena cava using pro-coagulation tube. Serums were immediately separated from blood by refrigerated centrifuge at 3000 rpm for 15 min (Anhui USTC Zonkia Scientific Instruments Co., Ltd, Anhui, China). Kidneys were removed for later histological analyses. SCr and BUN were performed using an automated biochemical analyzer (c8000A-701, Roche Diagnostics, German). Serum NGAL was quantitatively measured using an NGAL ELISA Kit (Bioporto, Denmark). The detailed operations were performed basing the manufacturer’s protocols. The absorbance values of the wells were read at 450 nm in a microplate reader (ANTHOS2010, England). The concentrations of NGAL were determined using a standard curve.

### 2.5 Histological analysis of renal tissues

Kidney issues were fixed in a 10% Formalin solution for later histological assessment. The issues were then dehydrated in ethanol, and embedded in paraffin wax. Four micrometer thick sections were obtained using a microtome. After that, they were stained with hematoxylin and eosin (H&E) for further histological examination.

#### 2.5.1 Statistics

The data were analyzed using SPSS 21.0. For data which followed a normal distribution, values were expressed as mean ± SD. Skewed data were expressed as median (25–75%) quartiles. Comparisons for parametric data among groups using a one-way analysis of variance (ANOVA), following LSD multiple comparisons. For non-parametric data, significance levels were from Kruskal–Wallis ANOVA, following by the *post hoc* Steel–Dwas test for multiple comparisons. A value of *p* < .05 was considered statistically significant.

## 3. Results

### 3.1 General exercise performance

During the experiment, one rat in Group EHS + Water refused to exercise, so it was removed. One rat in Group EHS + Water and two rats in Group EHS + ORS died 1 h after the onset of heatstroke, their final Tc was 42.2 °C, 42.8 °C, and 42.1 °C, respectively. At the end of the experiment, all rats’ core temperatures exceeded 40.5 °C, and almost all of them were unable to run, with belly clinging to the runway. As shown in Table 1, the exercising time in Group EHS was shorter than Group EHS + Water (105.77 ± 7.061 min VS 119.92 ± 13.18 min, *p* = .008). The time in Group EHS + ORS was 132.92 ± 15.89 min, higher than that in Group EHS (*p* < .001). Final Tc in Group EHS was 41.60 ± 0.69 °C, almost the same as that in Group EHS + Water and Group EHS + ORS, the difference didn’t reach statistical significance (*p* = .727, *p* = .962). The water loss percentage of Group EHS + Water (9.38 ± 1.52%) was not significantly different from Group EHS (8.78 ± 2.42%) (*p* = .255). In Group EHS + ORS, the percentage (8.78 ± 2.42) was no significant from Group EHS (*p* = .807).

**Table 1.** The results of exercising time, final Tc, and weights.

Groups	Exercising time (min)	Final Tc (°C)	Weights (pre) (g)	Weights (post) (g)	Water loss (%)
Control	–	36.17 ± 3.03	248.57 ± 16.54	239.86 ± 15.90	3.49 ± 1.07
EHS	105.77 ± 7.061	41.60 ± 0.69	231.95 ± 8.17	211.97 ± 8.39	8.62 ± 1.20
EHS + Water	119.92 ± 13.18**	41.37 ± 0.77	224.83 ± 9.70	203.70 ± 8.44	9.38 ± 1.52
EHS + ORS	132.92 ± 15.89***	41.57 ± 0.75	226.62 ± 10.21	206.78 ± 12.06	8.78 ± 2.42

Values are expressed as mean ± SD.

In contrast with Group EHS, *p < .05, **p < .01, ***p < .001.

### 3.2 Traditional kidney biomarkers

**Figure 1.**
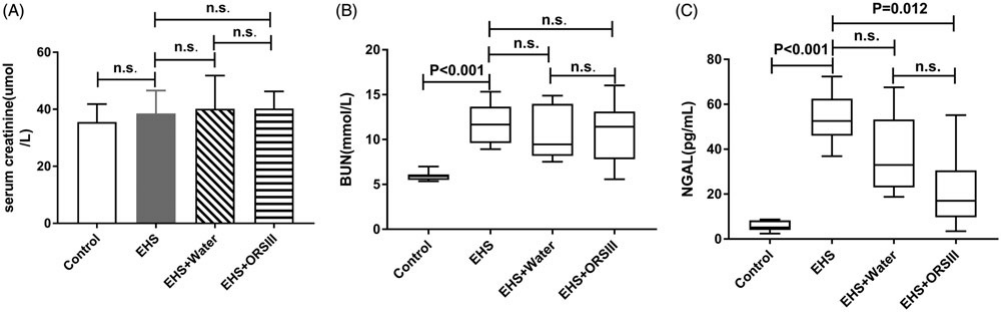
Concentrations of SCr, BUN, and NGAL in Group Control, EHS, EHS + Water and EHS + ORS. (A) values are mean ± SD, significance levels were from one-way ANOVA following LSD multiple comparisons. (B–C) values are median(25%–75%), significance levels were from Kruskal–Wallis ANOVA, following *post hoc* Steel–Dwas test.

For the SCr level, there is no difference among groups (*p* > .05). The serum urea levels in Group EHS were significantly higher than Group Control (*p* < .001). However, no statistically significant difference was found between Group EHS and Group EHS + Water, as well as Group EHS and Group EHS + ORS. The results were depicted in Figure 1.

### 3.3 Novel biomarker NGAL

The NGAL level in Group EHS was 52.55 (46.05–62.51) pg/ml, almost 10 times higher than Group Control, in which NGAL was 5.19 (4.06–8.33) pg/ml (*p* < .001). Similarly, the level in Group EHS + ORS was17.04 (9.73–30.56) pg/ml, lower than Group EHS (*p* = .012), however, no statistically significant difference was found between Group EHS and Group EHS + Water (*p* > .05).

### 3.4 Histological changes

**Figure 2.**
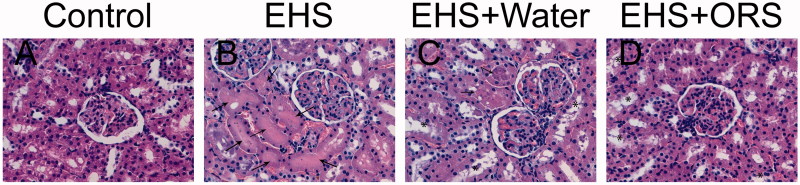
Representative renal histopathologies of rats (H&E, ×400). (A) Normal kidney, both the glomerus and tubular were presented with a normal microscopic appearance. (B) A typical kidney from Group EHS, showing flattened lumens filled with eosinophilic materials (↑). (C) Kidney from Group EHS + Water, the injured tubules showed degeneration of the tubular epithelium and sloughing of the brush border membrane (*), some lumens were also filled with eosinophilic materials (↑). (D) Kidney from Group EHS + ORS, the tubular injury was milder (*).

Under light microscope, both the glomerulus and tubules in Group Control showed a normal microscopic appearance (Figure 2(A)). In Group EHS, Group EHS + Water and Group EHS + ORS, the tubular damage was severer compared with glomerular injury, especially in proximal tubules. Moreover, degeneration of the tubular epithelium and sloughing of the brush border membrane were observed in Group EHS + ORS (Figure 2(D)), while in Group EHS and Group EHS + Water, the proximal tubular damage was much severer – the tubular atrophy with flattened lumens filled with eosinophilic materials (Figure 2(B,D)). No presence of neutrophil or other inflammatory cells infiltrating glomeruli and tubular epithelia.

## 4. Discussion

Along with the worldwide heat wave, the morbidity and mortality of EHS rise rapidly, which brings a great loss to society and individuals. Exposing to a hot environment for a long time, especially for those work in the outdoor, will easily raise the risk of EHS. Our results suggested that ORS III pretreatment accounted for improvements in exercise tolerance. The final Tc in EHS + ORS Group was almost the same as that in EHS + Water Group, which was inconsistent with our original assumption. That might because the exercising time in Group EHS + ORS was prolonged, and heat kept accumulating with the time, which offset the effect of ORS III, leading to a slightly higher core temperature in Group EHS + ORS. In addition, since ORS III prevented rats from losing too much water during the exercise, it helped rats keep rehydrated, so the water loss percentage in Group EHS + ORS was less than group EHS + Water. We assumed that there would be a significance between this two groups if the sample size was increased. Noticeably, one rat in Group C and two rats in Group D died one hour after the experiment, their core temperatures were 42.2 °C, 42.8 °C, and 42.1 °C, respectively. One study showed that once the Tc exceeded 42 °C, although active physical cooling and other supportive measures were made, the organ injuries kept aggravating, leading to multiple organ dysfunction syndrome(MODS), and even death [21]. We had to take more active measures to treat heatstroke once core temperature was over 42 °C.

We found that NGAL level reveals kidney damage earlier in the course of disease than the routine biomarkers. Creatinine is a byproduct of creatine phosphate in muscle, and is removed from the blood by the kidneys. It is the most commonly used indicator of renal function but easily influenced by high protein intake [22]. In addition, The serum creatinine level not only reflects renal excretion but also generation, intake and metabolism of creatine, so it does not estimate the glomerular filtration rate (GFR) adequately [23]. Since BUN is originally produced by liver and then eliminated by kidney, both kidney and liver dysfunction would affect the circulating concentration of BUN [24]. Research has shown that heatstroke would result in MODS [25], which meant that, under such circumstance, BUN could not reflect kidney damage accurately. What’s more, BUN starts to rise only when the GFR decreased to 30% of normal [26]. Therefore, early diagnosis of renal injury could be challenging using those routine biochemical parameters. We found that circulating levels of SCr and BUN were of no significance between Group B and Group C, as well as Group B and Group D, which demonstrated that the traditional clinical kidney biomarkers were not sensitive enough to detect the kidney injury [8]. But unlike those biomarkers, NGAL was more sensitive to find the early kidney lesions [15]. It was also worth mentioning that the concentration of NGAL was positively correlated with the severity of heatstroke [24]. Thus, kidney injury in Group EHS was severer than Group EHS + Water and Group EHS + ORS, which was consistent with the kidney pathology change found in our experiment. Notably, no difference was found between Group EHS and Group EHS + Water, which meant that water showed no benefit in protecting kidney from heatstroke. This finding was consist with what Von Duvillard et al. found, who suggested that athletes should take fluids containing electrolytes rather than plain water when exercises lasting longer than 1 h [27]. NGAL promotes the early detection of kidney injury and may suggest severity of kidney injury in EHS rats.

Some heatstroke induced pathological changes in kidney were associated with the influence of both exercise and hyperemia [24]. In our study, EHS induced kidney injury mainly located in tubules, while the glomerular pathological changes were not very prominent. One reason was that intensive exercising in hot weather led to water loss, which further aggravated the kidney ischemic damage. Greater ischemia damage led to proximal tubular brush border membrane sloughing [7]. Another reason was that hyperthermia could cause proximal tubular epithelial cellular dysfunction [28]. However, our findings were contrary to what Segev et al. found – their research showed that kidney damage in heatstroke was not limited to the renal tubules but also involved glomeruli [24]. More deep research should be made to locate the lesion specifically and accurately.

Fluid and supplementation for heatstroke were extremely important. Even just 1% reduction due to water loss in body weight would increase plasma osmolality, and would cause the intracellular and extracellular electrolyte imbalance [29]. Hypohydration not only increased physiologic strain, decreased exercise, but also caused heat acclimation [19]. The possible kidney protective mechanisms of ORS III were in the following ways: firstly, in clinical practice, oral rehydration therapy had proved to be effective [13]. Intake of ORS III was a good way to replace the fluid-losing during exercise, and sufficient water could improve kidney perfusion, which reduced the ischemic damage in kidney. Secondly, ORS III would promote water and sodium absorption due to its optimal concentration of components. The common ion disorders for heatstroke patients were hypokalemia, hypophosphatemia, hyponatremia, hypocalcemia, and hypomagnesemia [6]. Electrolyte imbalance would further result in ATP pump dysfunction, increasing the cell membrane permeability, and led to swelling of the renal tubular epithelial cell, accumulation of toxic metabolites, promoting apoptosis of the cells [7,30]. ORS III could protect kidney from ion imbalance. Thirdly, the oral solution was beneficial to improving the damage of temperature regulation caused by heat. Finally, sodium citrate, a component in ORS III, is also an effective bicarbonate analog after being metabolized. Since bicarbonate has proved to prevent kidney injury through lowering uric acid levels in heatstroke [31,32], citrate may play a role in preventing AKI. ORS III is crucial to and will benefit EHS rats.

A key limitation of our study was that the pathological changes reported were based on light microscopy, which was unable to detect glomerular injury accurately. Moreover, the current findings were only applicable to the male rats. Since the majority of exertional heatstroke cases were reported in men, it remained to be seen whether women demonstrated similar responses after drinking ORS III.

Heatstroke is a preventable disease, and there are two main therapeutic methods in patients with heatstroke: physical cooling and support of organ-system function [1]. As for the prevention, people can acclimatize themselves to heat, reduce activity levels, drink additional water, and prolong the time they spend in air-conditional rooms [33]. Although avoidance of dehydration and ion imbalance was effective in preventing heatstroke [34], no oral solution therapies or drugs had yet recommended. Given the evidence that heatstroke may be one of the factors causing CKD in tropical regions [5], and AKI is related to CKD [35], we highly recommend clinical trials to test whether pretreatment of ORS might prevent AKI or mitigate the CKD progression in those areas. Our study offered an insight into prevention of heatstroke and extended the application of ORS III other than treating infant diarrhea.

## Conclusion

This study demonstrated that EHS could lead to kidney injury in rats, and pretreatment of ORS III could alleviate the kidney injury in EHS rats, while water showed no benefit in prevention of kidney injury. NGAL, a novel kidney injury biomarker, was more accurate in assessing kidney damage compared with traditional parameters such as SCr and BUN. Kidney damage in EHS was mainly limited to renal tubules in an early stage, while glomerular injuries were rarely seen. From a practical standpoint, these findings suggest that clinicians should take note of precautionary measures of drinking oral solution before exercising in extreme weather. Early diagnosis of kidney damage, with early intervention, may prevent overt failure and potentially decrease mortality.
